# Validity of moyers mixed dentition analysis for Saudi population

**DOI:** 10.12669/pjms.316.8377

**Published:** 2015

**Authors:** Yousef H. Al-Dlaigan, Nasser D. Alqahtani, Khalid Almoammar, Thikriat Al-Jewair, Fahad Bin Salamah, Mohamme Alswilem, Sahar F. Albarakati

**Affiliations:** 1 Yousef H. Al-Dlaigan, BDS, MS, Cert Pedia, PhD. College of Dentistry, King Saud University, Riyadh, Saudi Arabia; 2 Nasser D. Alqahtani BDS, MS, FRCD. College of Dentistry, King Saud University, Riyadh, Saudi Arabia; 3 Khalid Almoammar BDS, MSc, MORTH, RCSed, PhD. College of Dentistry, King Saud University, Riyadh, Saudi Arabia; 4 Thikriat Al-Jewair, BDS, MBA, MSc, MS, FRCD(C). College of Dentistry, University of Dammam, Saudi Arabia; 5 Fahad Bin Salamah, BDS. Intern, College of Dentistry, King Saud University, Riyadh, Saudi Arabia; 6 Mohammed Alswilem, BDS. Intern, College of Dentistry, King Saud University, Riyadh, Saudi Arabia; 7 Sahar F. Albarakati, BDS, MS, Cert Ortho. Department of Pediatric Dentistry and Orthodontics, College of Dentistry, King Saud University, Riyadh, Saudi Arabia

**Keywords:** Moyers probability tables, Mixed dentition analysis

## Abstract

**Objectives:**

To evaluate the applicability of Moyers probability tables and to formulate more accurate mixed dentition prediction tables in the Saudi population.

**Methods:**

A cross-sectional study was conducted at the College of Dentistry, Kind Saud University, Saudi Arabia. The data were collected from 410 (203 males and 207 females) orthodontic study models, which had erupted mandibular permanent incisors, maxillary, mandibular canines and premolars. The mesiodistal widths were measured using a digital caliper with an accuracy of 0.01 mm. Student’s paired t-test was used to compare the mean width values derived from this study with the values derived using the Moyers table. Simple linear regression was used to evaluate the linear relationship between the combined mesiodistal widths of the mandibular permanent incisors and the canine-premolar segments in each dental arch.

**Results:**

The regression equations for the maxillary canine-premolar segment (males: Y=10.27+0.48X; females: Y=11.71 + 0.39X) and the mandibular canine-premolar segment (males: Y=9.71 + 0.40X; females: 11.28 + 0.39X) were used to formulate new probability tables on the Moyers pattern. Statistically significant differences were observed between predicted widths in our subjects and the widths obtained using Moyers tables.

**Conclusions:**

The new prediction tables derived in this study provided a more precise mixed dentition space analysis than Moyers prediction tables in estimating tooth dimensions in the Saudi population.

## INTRODUCTION

Arch length discrepancy is defined as the difference between the amount of dental arch space that is available and the amount of tooth size that needs to be accommodated.^1^ Measuring arch length discrepancy in children and adolescent who are in the mixed dentition stage is challenging. Therefore, mixed dentition analysis is normally done to predict the space required for the alignment of the unerupted canines and premolars since the space available between the lateral incisor and the first permanent molar is limited.^2^ This analysis is based not only on the correlation between the sizes of all permanent teeth anterior to the first molars, but also on the available arch perimeter, and the expected changes in arch dimensions resulting from growth and development.^3^

There are three main approaches for the prediction of the width of unerupted permanent canines and premolars. Direct measurement on the radiograph, regression equations and tables, and combination of both.^2^,^4^-^7^ The correlational statistical methods are most frequently applied because of their simplicity. Moyers was the first researcher to predict the widths of the permanent canines and premolars using the sum of the lower permanent incisors by using probability tables.^8^

Several small studies were conducted to predict the mesiodeistal widths of permanent canines and premolars in the Saudi population.^9^-^12^ It has been reported that Saudis have smaller teeth than population of northern European descents by observing the actual measurements of the sample which followed Moyers charts at 35% confidence interval rather than the commonly used 75%.^10^ Another study formulated regression equations for Saudi samples and concluded that the 50% level is a more accurate determinant for canines and premolars when both sexes are combined.^12^ The studies showed different results.

Periodical validation of mixed dentition space analyses is required in each generation due to discrepancies of facial characteristics and in sizes of tooth.^13^ Therefore, this study was carried out to derive new probability tables, to predict equations for the Saudi population to perform mixed dentition space analysis more accurately, and to evaluate the accuracy of the Moyers tables method on Saudi population.

## METHODS

A total of 410 pairs of randomly selected dental casts (203 males and 207 females) were collected from different clinics in the college of dentistry, at King Saud University, Riyadh, Saudi Arabia. The average age of the subjects was 15.10 ± 0.45 years and the range was 13-20 years. The study was approved by the College of Dentistry Research Center, King Saud University. Appropriate sample size was established; α=0.05 and 1-β=0.95 with estimated standard deviation of 1.2mm, at least 360 patients should be included.

The mesiodistal widths of the lower incisors, the upper and lower canines and premolars were measured on each cast. In order to minimize the impact of human error, only one examiner recorded all measurements using an electronic digital caliper (Mitutoyo Manufacturing Co. Ltd., Tokyo, Japan) with an accuracy of 0.01mm. The caliper beaks were inserted labially parallel to the occlusal surface, then the distance between the contact points between the proximal surfaces was measured.^11^ The inclusion criteria were defined as:

1. Native Saudis.
2. Presence of fully erupted upper and lower premolars and canines, as well as lower incisors.
3. Teeth to be measured must not have any proximal restoration.
4. Measurement must be done on a pre-orthodontic treatment models.
5. No congenitally missing, malformed, broken or chipped teeth.

All subjects with malocclusion or crowding were excluded. To assess intra-examiner reliability of the measurement, 20 pairs of dental casts were randomly selected and re-measured by the same examiner with a one-week interval. The paired-sample t-test indicated no statistically significant difference between the first and the second readings (p>0.1 and standard errors ≤ 0.004). Pearson’s correlation coefficient indicated substantial correlation between the first and the second readings (r=0.96).

### Statistical analysis

The data were analyzed using SPSS version 21.0 for windows (statistical package for social sciences, IL, USA). Descriptive statistics and Pearson’s correlation coefficient were used. A standard simple linear regression equation of the form Y= a + b(X) was derived to evaluate the linear relationship between combined mesiodistal widths of the mandibular permanent incisors (X) and the mesiodistal widths of the canine-premolar segments(Y) in the upper and lower arches. Regression estimates and correlation values were assessed using student’s t-test. Student’s paired t-test was used to compare the mean width values derived from this study with the width values derived using Moyers table. A p-value of <0.05 and 95% confidence intervals were used to report the statistical significance and precision of the estimates.

## RESULTS

The mean widths of the mandibular incisors, the sum of the mandibular and the maxillary canine-premolar segments are presented in Table-I. There was highly statistically significant positive correlation between the mandibular incisors and the sum of the maxillary canine-premolar and mandibular canine-premolar segments for all subjects (r= 0.55 and 0.53 respectively; p<0.0001). A similar finding was noted when the data were analyzed by gender (male: r=0.61 and 0.60; p<0.0001; female: r= 0.52 and 0.51; p<0.001).

**Table-I:** Mandibular incisors, Mandibular Canine Premolars, and Maxillary Canine Premolars teeth width of Study subjects (n=410).

Variables	Minimum	Maximum	Mean	Standard Deviation
Mandibular Incisors	15.91	26.22	21.50	1.63
Mandibular Canine Premolars RT	15.84	24.69	19.88	1.38
Mandibular Canine Premolars LT	16.62	23.36	20.04	1.33
Mandibular Canine Premolars(Average)	16.23	24.03	19.96	1.30
Maxillary Canine Premolars RT	16.60	24.53	20.20	1.26
Maxillary Canine Premolars LT	16.20	24.04	20.35	1.36
Maxillary Canine Premolars (Average)	16.57	24.29	20.27	1.25

For all subjects, the regression coefficient for the maxillary canine-premolar segment was 0.42 and for the segment it was 0.43. For the male subjects it was 0.48 and 0.49 respectively and for females it was 0.39 and 0.39. All these estimates were highly statistically significant (p<0.001) indicating a positive linear relationship. The standard error of these estimates ranged from 0.032 to 0.046. The values of r2 indicate the proportional variability in the width values of maxillary and mandibular canine and premolars as explained by the mandibular incisors (Table-II).

**Table-II:** Regression equation and estimates for the prediction of maxillary and mandibular canine and premolars teeth width of study subjects.

Subjects	Dependent Variables	a	b	SEE of ‘b’	p-value	r	r ^2^	Regression Equation
All	∑ Max.CPM	11.22	0.42	0.032	<0.0001	0.55	0.30	Y=11.22+0.42(X)
	∑ Mand.CPM	10.75	0.43	0.033	<0.0001	0.53	0.29	Y=10.75+0.43(X)
Male	∑ Max.CPM	10.27	0.48	0.044	<0.0001	0.61	0.37	Y=10.27+0.48(X)
	∑ Mand.CPM	9.71	0.49	0.046	<0.0001	0.60	0.36	Y=9.71+0.49(X)
Female	∑ Max.CPM	11.71	0.39	0.044	<0.0001	0.52	0.27	Y=11.7+ 0.39(X)
	∑ Mand.CPM	11.28	0.39	0.045	<0.0001	0.51	0.26	Y=11.28+0.39(X)

∑ Max CPM = sum of width of the maxillary canine and premolars; ∑ Mand CPM= sum of width of mandibular canine and premolars; a = constant; b= estimate; SEE = standard error of estimate; r= correlation coefficient; r2 = coefficient of determination; X= Mandibular incisors 32,31,41,42.

The differences between the regression values in this study and the Moyers probability tables at different percentile levels the maxillary and mandibular canine-premolar segments are shown in Tables III and IV. At all the percentile levels, the width values derived using Moyers probability tables were statistically significantly higher than the width values derived using the regression equations in our study.

**Table-III:** Differences between the regression values of this study and from the Moyers probability tables at different percentile levels in Male and Female subjects for Maxillary Canine-premolar segments.

Percentile	Difference Y1-Y2 (mm) Maxillary Canine Premolar segments							
	Male				Female			
	Mean difference	t-value	p-value	95% Confidence interval of the difference	Mean difference	t-value	p-value	95% Confidence interval of the difference
5	-0.022	-1.91	0.088	(-0.05,0.004)	-0.296	-6.13	<0.0001	(-0.40,-0.19)
15	-0.099	-8.31	<0.0001	(-0.12,-0.07)	-0.183	-8.33	<0.0001	(-0.23,-0.14)
25	-0.148	-13.56	<0.0001	(-0.17,-0.12)	-0.127	-7.95	<0.0001	(-0.16,-0.09)
35	-0.179	-18.14	<0.0001	(-0.19,-0.16)	-0.079	-5.32	<0.0001	(-0.11,-0.05)
50	-0.223	-23.51	<0.0001	(-0.24,-0.20)	-0.015	-1.12	0.265	(-0.04,0.01)
65	-0.260	-27.47	<0.0001	(-0.28,-0.24)	0.035	2.58	0.011	(0.008,0.06)
75	-0.284	-29.88	<0.0001	(-0.30,-0.26)	0.070	5.10	<0.0001	(0.043,0.09)
85	-0.307	-31.69	<0.0001	(-0.32,-0.29)	0.130	7.36	<0.0001	(0.075,0.14)
95	-0.333	-32.01	<0.0001	(-0.35,-0.31)	0.136	9.45	<0.0001	(0.10,0.16)

Y1= Predicted mesiodistal width of canine-premolar segments in this study; Y2= predicted mesiodistal width of canine-premolar segments from the Moyers tables.

**Table-IV:** Differences between the regression values of this study and from the Moyers probability tables at different percentile levels in Male and Female subjects for Mandibular Canine-premolar segments.

Percentile	Difference Y1-Y2 (mm) Maxillary Canine Premolar segments							
	Male				Female			
	Mean difference	t-value	p-value	95% Confidence interval of the difference	Mean difference	t-value	p-value	95% Confidence interval of the difference
5	-0.439	-96.43	<0.0001	(-0.45,-0.43)	0.235	10.96	<0.0001	(0.18,0.28)
15	-0.453	-160.49	<0.0001	(-0.46,-0.44)	0.166	13.73	<0.0001	(0.14,0.19)
25	-0.464	-185.19	<0.0001	(-0.47,-0.45)	0.125	12.63	<0.0001	(0.10,0.14)
35	-0.471	-210.79	<0.0001	(-0.42,-0.46)	0.091	9.49	<0.0001	(0.07,0.11)
50	-0.481	-224.84	<0.0001	(-0.49,-0.47)	0.047	4.99	<0.001	(0.03,0.06)
65	-0.489	-228.39	<0.0001	(-0.49,-0.48)	0.011	1.20	0.231	(-0.01,0.03)
75	-0.494	-231.80	<0.0001	(-0.50,-0.49)	-0.013	-1.38	0.17	(-0.03,0.06)
85	-0.499	-231.15	<0.0001	(-0.50,-0.49)	-0.035	-3.71	<0.0001	(-0.05,-0.02)
95	-0.506	-214.43	<0.0001	(-0.51,-0.50)	-0.058	-5.90	<0.0001	(-0.08,-0.04)

Y1= Predicted mesiodistal width of canine-premolar segments in this study; Y2= predicted mesiodistal width of canine-premolar segments from the Moyers tables

For different width values of mandibular incisors (from 19.5 to 25.0), the predicted width of the maxillary canine and premolars & mandibular canine and premolars from this study are given in Table-V along with the Moyers table values at the 35th, 50th and 65th percentiles.

**Table-V:** Predicted width values of the maxillary and mandibular canine-premolar segments by each Moyers table value in the study subjects.

Mandibular	This Study		Moyer’s values					
Incisors	Predicted width	Predicted width	Maxillary width			Mandibular width		
32324142	Maxillary Canine Premolars	Mandibular Canine Premolars	35%	50%	65%	35%	50%	65%
19.5	19.21	18.78	19.6	20.0	20.4	19.0	19.4	19.8
20.0	19.63	19.21	19.9	20.3	20.6	19.3	19.7	20.1
20.5	19.81	19.40	20.2	20.6	20.9	19.6	20.0	20.4
21.0	20.04	19.64	20.5	20.8	21.2	19.9	20.3	20.7
21.5	20.25	19.86	20.8	21.1	21.5	20.2	20.6	21.0
22.0	20.45	20.07	21.0	21.4	21.8	20.5	20.9	21.3
22.5	20.65	20.27	21.3	21.7	22.0	20.8	21.2	21.6
23.0	20.87	20.50	21.6	21.9	22.3	21.1	21.5	21.9
23.5	21.08	20.71	21.9	22.2	22.6	21.4	21.8	22.2
24.0	21.27	20.92	22.1	22.5	22.8	21.7	22.1	22.5
24.5	21.47	21.13	22.4	22.8	23.1	22.0	22.4	22.8
25.0	22.09	21.78	22.7	23.0	23.4	22.3	22.7	23.1

## DISCUSSION

Mixed dentition space analysis is the most commonly used method in the diagnosis and planning of treatment in young subjects. The available prediction tables are derived from Caucasian populations, and thus their use in other populations lack accuracy. In order to achieve the ideal over jet, overbite and occlusion for the Saudi patients, we need to have accurate predictions of their teeth sizes. Accurate analysis is particularly important in cases where the treatment plan might include space management, serial extraction, guidance of eruption and periodic observation.^14^

As the sum of the mandibular incisors and maxillary and mandibular posterior segments are linearly related, many studies had developed linear regression equations to use for their specific populations.^10^,^15^-^18^ For the Saudi population, new regression equations were derived for both males and females. The correlation coefficients (0.51 to 0.61) obtained in this study are similar to the coefficients of a study by Asiry,^11^ but slightly higher than the values reported from two other studies.^6^,^7^ The coefficients of determination, that indicated the predictive accuracy of the regression equations were between 0.26 to 0.37. This suggested that 26% to 37% of the change in the canine-premolar widths were explained by knowing the combined mandibular incisors widths. Our estimates are smaller than findings of Arslan^5^ but comparable to the values reported by Jaiswal^19^ and Jaroontham.^20^

Previous studies have confirmed that different races and ethnic groups present with different teeth sizes.^6^,^14^,^21^ Our study showed that Saudis have smaller teeth compared to the northern European populations. Comparison of the values showed statistically significant differences between the predicted mesiodistal tooth widths in our study and that of Moyers probability tables in male subjects at all percentiles; whereas no significant differences were observe for the female subjects at the 50% percentile for the maxillary canine-premolars and at the 65% and 75% percentiles for the mandibular canine-premolar segments.

From our analysis it can be stated that Moyers tables tend to either overestimate or underestimate the mesiodistal canine-premolars widths in our population. Previous validity studies of Moyers tables also showed similar patterns.^22^ An earlier study on the Saudi population found that the recommended 75% confidence level of the Moyers probability tables overestimated the sizes of Canines and premolars.^10^ Our results follow Moyers charts more closely at the 35% confidence interval which is similar to those found by Alkhadra.^12^ Comparing teeth sizes of Saudis to other populations revealed that Saudis have smaller teeth than Malai, Jordanian, Turkish and Egyptian populations.^5^,^6^,^23^,^24^

The decision to treat or not to treat and the type of treatment are based on the results of mixed dentition space analysis. It was previously observed that Moyers tables are accurate when applied exclusively to white patients.^25^ Moreover from the Moyers prediction tables it is not possible to find the raw numbers for the measurement of all the teeth that had been included in his study.^8^

Earlier studies that have investigated the measurements of the Saudi’s dentition were limited due to the small number of subjects included.^10^,^12^ This study tried to incorporate a bigger sample size within the inclusion criteria to overcome the limitation of the small sample size. The sample for this study was taken from a pool of patients attending the dental clinics from college of dentistry in King Saud University, therefore, the results may not be generalizable to the Saudi population in different age groups or settings.

## CONCLUSION

Saudis have smaller teeth when compared to Moyers prediction table. The new prediction tables can be used to accurately assess the space condition of teeth in each patient, which will make the decision easier for orthodontists in the region on whether to extract or maintain space in a child to avoid potential crowding.
